# Dual CD47 and PD‐L1 blockade elicits anti‐tumor immunity by intratumoral CD8^+^ T cells

**DOI:** 10.1002/cti2.70014

**Published:** 2024-11-22

**Authors:** Susan N Christo, Keely M McDonald, Thomas N Burn, Nadia Kurd, Jessica Stanfield, Megan M Kaneda, Ruth Seelige, Christopher P Dillon, Timothy S Fisher, Bas Baaten, Laura K Mackay

**Affiliations:** ^1^ Department of Microbiology and Immunology The University of Melbourne at the Peter Doherty Institute for Infection and Immunity Melbourne Australia; ^2^ Oncology Research Unit Pfizer Inc. San Diego CA USA

**Keywords:** adaptive immunity, CD47, CD8^+^ T cells, immune checkpoint blockade, immunotherapy, tumor‐resident CD8^+^ T cells

## Abstract

**Objectives:**

Bispecific antibodies targeting CD47 and PD‐L1 (CD47 × PD‐L1 BisAb) demonstrate efficacy against a range of solid cancers. While dual blockade negates anti‐CD47‐mediated toxicity, the effect of combined innate and adaptive immune activation on protective tumor‐resident CD8^+^ T cells has yet to be fully elucidated.

**Methods:**

CD8^+^ T cell populations were tracked upon CD47 × PD‐L1 BisAb treatment in an orthotopic model of murine breast cancer where anti‐tumor immunity is mediated by CD8^+^ T cells. Immune responses were also compared with anti‐PD‐L1 monotherapy to assess the advantage of dual checkpoint targeting.

**Results:**

We found that CD47 × PD‐L1 BisAb treatment augmented CD8^+^ T cell responses in tumors, which resulted in enhanced tumor control. Compared with anti‐PD‐L1 treatment, dual CD47 and PD‐L1 blockade promoted greater numbers of antigen‐specific tumor‐resident CD8^+^ T cells that exhibited increased cytokine production.

**Conclusions:**

Engagement of innate and adaptive immune checkpoint molecules via CD47 × PD‐L1 BisAb treatment resulted in robust CD8^+^ T cell responses, including the induction of tumor‐resident CD8^+^ T cells that exhibited functionally superior anti‐tumor immunity. These results demonstrate that innate immune activation potentiates anti‐tumor adaptive responses, highlighting the use of dual checkpoint blockade as an optimal strategy for promoting CD8^+^ T cell‐mediated protection.

## Graphical abstract

Bispecific antibodies targeting CD47 and PD‐L1 (CD47 × PD‐L1 BisAb) demonstrate efficacy against a range of solid cancers, but the effect of combined innate and adaptive immune activation on protective tumor‐resident CD8^+^ T cells has yet to be fully elucidated. We found that CD47 × PD‐L1 BisAb treatment resulted in robust CD8^+^ T cell responses, including the induction of tumor‐resident CD8^+^ T cells that exhibited functionally superior anti‐tumor immunity when compared to anti‐PD‐L1 monotherapy. These results demonstrate that innate immune activation potentiates anti‐tumor adaptive responses, highlighting the use of dual checkpoint blockade as an optimal strategy for promoting CD8^+^ T cell‐mediated protection.

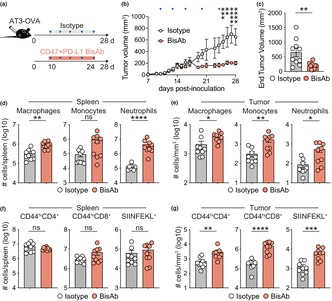

## Introduction

Immune checkpoint blockade (ICB) has revolutionised the approach to treating cancer.^1^, ^2^, ^3^ Among ICB therapies, those targeting the PD‐1/PD‐L1 axis have been extensively characterised and shown to curtail tumor growth by promoting CD8^+^ T cell function.^4^, ^5^ However, the efficacy and longevity of these therapies are not universal, with poor intratumoral T cell influx and low tumor immunogenicity contributing to the reduced success of ICB in patients with solid cancers.^6^ Therefore, combined ICB regimens present a viable avenue for overcoming the current resistance to monotherapies.

Innate immune checkpoints are increasingly being explored as strategies to promote anti‐tumor immunity.^7^ CD47 is an attractive target as it is highly expressed by tumor cells^8^, ^9^ and macrophages, and acts as a ‘don't eat me’ signal.^9^ Disrupting the CD47/SIRPα axis permits macrophage‐mediated phagocytosis, which may eliminate malignant cells and improve antigen presentation.^8^, ^9^, ^10^, ^11^, ^12^ Indeed, anti‐CD47 antibody treatments are effective at reducing tumor burden and have been recently found to affect CD8^+^ T cell function by enhancing dendritic cell (DC) and macrophage activation.^11^, ^13^, ^14^, ^15^ Moreover, anti‐CD47 therapies have only demonstrated efficacy in the presence of CD8^+^ T cells, suggesting direct cross‐talk among immune populations.^12^ However, anti‐CD47 antibody monotherapies may induce significant clinical side effects, including erythrotoxicity and anaemia.^10^ To circumvent the high affinity binding of anti‐CD47 to red blood cells, next‐generation therapeutics have been designed that preferentially target CD47 on cells expressing PD‐L1. Here, we utilised CD47 × PD‐L1 BisAb that has been engineered with reduced affinity towards CD47 to negate erythrocyte binding, while retaining engagement with PD‐L1‐expressing cells in the tumor.^16^ Indeed, we demonstrated that this method of dual targeting CD47 and PD‐L1 could induce an increase in myeloid cells and T cells into tumors, along with enriched type I IFN signalling and antigen presentation.^16^ Additionally, gene analyses of immune cell infiltrates revealed increased transcriptional signatures associated with stem‐like and effector CD8^+^ T cells.^16^ However, the influence of dual innate and adaptive checkpoint blockade on the formation and function of tumor‐resident CD8^+^ T cell populations has not been explored.

Reinvigorating CD8^+^ T cell functionality by reversing inhibitory receptor (IR) pathways is the goal of many ICB regimens. Notably, dysfunctional exhausted T cells are considered the main target of such therapies given their elevated expression of checkpoint molecules such as PD‐1 and CTLA‐4. However, when compared to these exhausted T cells, CD8^+^ tumor‐infiltrating lymphocytes (TIL) exhibiting a tissue‐resident T cell‐like phenotype have demonstrated increased functionality despite their high IR expression.^17^, ^18^ These tumor‐resident CD8^+^ TIL have been shown to remain anchored at sites of tumorigenesis where they provide rapid local protection^19^, ^20^ and have been associated with improved survival of patients with a range of solid cancers.^21^

Here, we aimed to elucidate intratumoral CD8^+^ T cell responses generated upon dual CD47 and PD‐L1 blockade in an orthotopic model of breast cancer. We found that CD47 × PD‐L1 BisAb resulted in robust anti‐tumor immunity by enhancing antigen‐specific tumor‐resident CD8^+^ TIL with superior cytokine production. Critically, CD47 × PD‐L1 BisAb could elicit greater CD8^+^ T cell responses than standard anti‐PD‐L1 monotherapy, suggesting that induction of myeloid cells may licence T cell functionality for durable responses. Overall, our findings demonstrate the mechanistic advantage of CD47 × PD‐L1 BisAb over standard ICB treatments and strengthens the clinical potential of this agent in currently ongoing trials (NCT04881045).

## Results

### CD47 × PD‐L1 BisAb treatment reduces AT3 tumor burden and bolsters intratumoral CD8^+^ T cells

**Figure 1:**
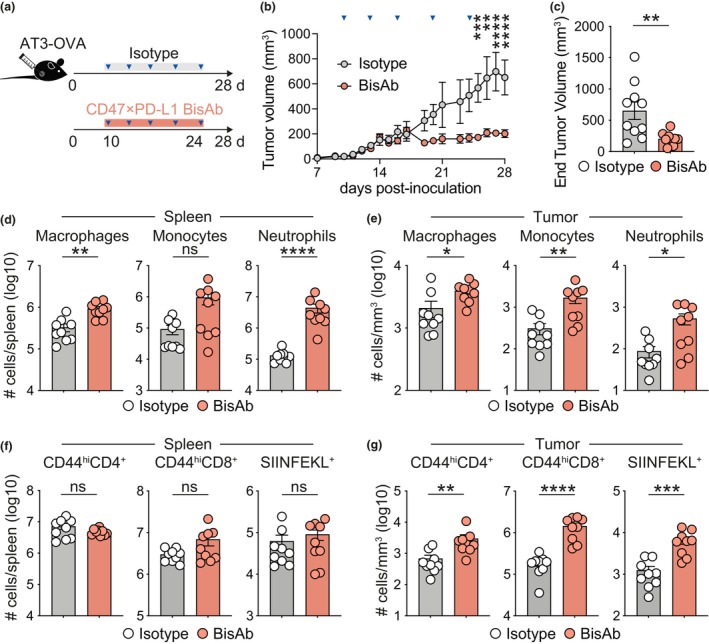
CD47 × PD‐L1 BisAb therapy reduces tumor burden and induces CD8^+^ T cell expansion. Mice were orthotopically inoculated with AT3‐OVA cells and treated with 40 mg kg^−1^ of isotype or CD47 × PD‐L1 BisAb i.p from Day 10 post inoculation every 3–4 days for a total of five injections before being assessed on Day 28. **(a)** Experimental schematic. **(b)** Tumor volume over time. **(c)** Tumor volume at experimental endpoint (Day 28). Enumeration of macrophages, monocytes and neutrophils in the **(d)** spleen and **(e)** tumor. Enumeration of polyclonal CD44^hi^CD4^+^ T cells, CD44^hi^CD8^+^ T cells and SIINFEKL^+^ CD8^+^ T cells in the **(f)** spleen and **(g)** tumor. Enumeration of cells in the tumor are expressed per mm^3^. The combined data of two independent experiments with a total of 9 or 10 mice per group is shown. ns, *P* > 0.05, *P ≤ 0.05, **P ≤ 0.01, ***P ≤ 0.001, ****P ≤ 0.0001, Mann–Whitney *U*‐test. Bars represent mean ± SEM, symbols represent individual mice.

While dual targeting of CD47 and PD‐L1 has been shown to control tumor growth,^16^ the breadth of intratumoral CD8^+^ T cell responses resulting from this treatment has not been characterised. Here, we employed an orthotopic model of triple‐negative breast cancer (AT3) expressing the model antigen ovalbumin (OVA; AT3‐OVA), which generates protective tumor‐resident CD8^+^ TIL that are necessary for tumor control.^19^ Importantly, we noted that AT3‐OVA cells expressed high levels of CD47 and PD‐L1 (Supplementary figure 1a), along with the expression of these molecules on macrophages and cDC populations isolated from the tumor and spleen (Supplementary figure 1b and c, and Supplementary figure 2). CD47 × PD‐L1 BisAb treatment of C57BL/6 mice inoculated with AT3‐OVA resulted in a significant reduction in tumor volume compared to mice treated with an isotype control antibody (Figure 1a–c). At 28 days post tumor inoculation, we found enhanced numbers of macrophages, monocytes and neutrophils in the spleen and tumor (Figure 1d and e), as well as an increased proportion of cDC1 compared to cDC2 populations upon CD47 × PD‐L1 BisAb therapy (Supplementary figure 3a and b), as previously reported.^16^

Upon quantifying the infiltration of CD44^hi^ T cells across organs, we did not observe significant alternations in CD44^hi^ CD4^+^ and CD8^+^ T‐cell numbers in the spleen and peritumoral mammary fat pad (MFP) of CD47 × PD‐L1 BisAb‐treated mice (Figure 1f and Supplementary figure 3c). However, we found significantly increased numbers of intratumoral CD4^+^ and CD8^+^ CD44^hi^ T cells after CD47 × PD‐L1 BisAb therapy (Figure 1g and Supplementary figure 3d). By leveraging the capacity to assess tumor antigen OVA‐specific SIINFEKL‐tetramer^+^ (SIINFEKL^+^) CD8^+^ T cell responses, we found that the majority of tumor‐reactive T cells were located within the tumor and to a much lesser extent within the spleen and MFP (Supplementary figure 3e and f), suggesting local antigen‐driven expansion. Indeed, SIINFEKL^+^ CD8^+^ T cells were significantly enhanced by CD47 × PD‐L1 BisAb treatment in the tumor compared to controls (Figure 1g) but were numerically unchanged in the spleen and MFP (Figure 1f and Supplementary figure 3c).

### CD47 × PD‐L1 BisAb treatment enhances tumor‐resident CD8^+^ TIL functionality

**Figure 2:**
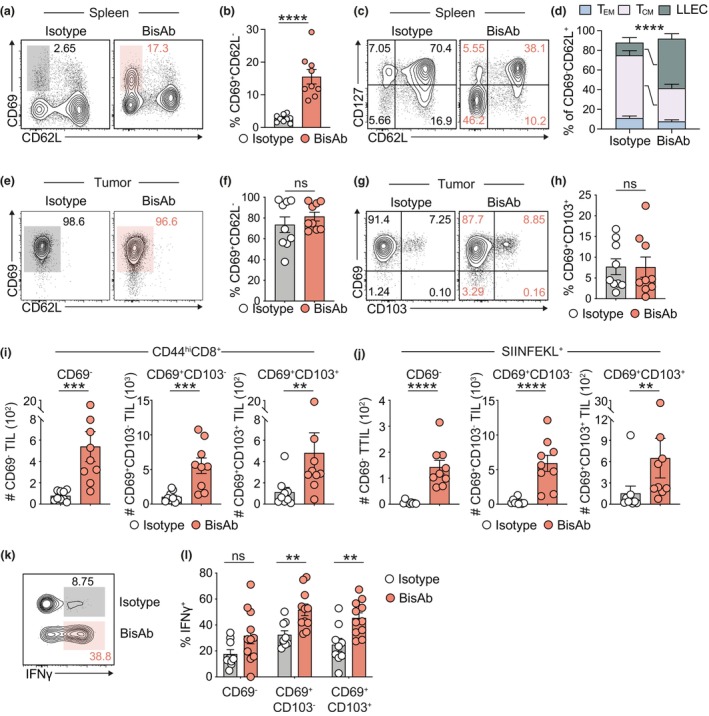
CD47 × PD‐L1 BisAb therapy enhances tumor‐resident CD8^+^ TIL formation and function. **(a–j)** AT3‐OVA‐bearing mice were treated with isotype or BisAb before being assessed on Day 28 post inoculation. **(a)** Representative contour plots and **(b)** frequency of CD69 and CD62L expression by CD44^hi^CD8^+^ T cells in the spleen. Shaded boxes in **(a)** represent the frequency of CD69^+^CD62L^−^ T cells. **(c)** Representative contour plots and **(d)** frequency of T_EM_ (CD127^+^CD62L^−^), T_CM_ (CD127^+^CD62L^+^) and LLEC (CD127^−^CD62L^−^) CD44^hi^CD8^+^ T cells in the spleen. **(e)** Representative contour plots and **(f)** frequency of CD69 and CD62L expression by SIINFEKL^+^ CD8^+^ T cells in the tumor. Shaded boxes in **(e)** represent the frequency of CD69^+^CD62L^−^ T cells. **(g)** Representative contour plots and **(h)** frequency of CD69 and CD103 expression by SIINFEKL^+^ CD8^+^ T cells in the tumor. Enumeration of **(i)** CD44^hi^CD8^+^ and **(j)** SIINFEKL^+^ CD69^−^, CD69^+^CD103^−^ and CD69^+^CD103^+^ TIL. **(k, l)** Intratumoral CD8^+^ T cells were stimulated with PMA/Ionomycin Day 18 post‐inoculation. **(k)** Representative contour plots of IFNγ expression by CD69^+^CD103^+^ TIL. **(l)** Frequency of IFNγ expression by CD69^−^ T_CIRC_, CD69^+^CD103^−^ and CD69^+^CD103^+^ TIL. Enumeration of cells in the tumor are expressed per mm^3^. The combined data of two independent experiments with a total of 9 or 10 mice **(b, d, f, g, h)** and 4–6 mice **(j)** per group is shown. ns, *P* > 0.05, **P ≤ 0.01, ***P ≤ 0.001, ****P ≤ 0.0001, Mann–Whitney *U*‐test. Bars represent mean ± SEM, symbols represent individual mice.

The CD8^+^ T cell pool comprises a range of populations with varied phenotype and functional capacities.^19^ We next sought to assess the impact of CD47 × PD‐L1 BisAb treatment on the breadth of CD8^+^ T cell populations generated in response to AT3‐OVA. While CD47 × PD‐L1 BisAb treatment did not appear to affect CD44^hi^CD8^+^ and SIINFEKL^+^ T‐cell numbers in the spleen (Figure 1f), we noted a striking shift in the T cell phenotype upon treatment. Specifically, we found that splenic CD8^+^ T cells exhibited elevated expression of the activation marker CD69 (Figure 2a and b) and observed decreased proportions of cells expressing a classical central memory T cell (T_CM_) phenotype (CD127^+^CD62L^+^) and increased generation of long‐lived effector T cells (LLEC; CD127^−^CD62L^−^) (Figure 2c and d). As expected, the vast majority of SIINFEKL^+^ CD8^+^ T cells in the tumor expressed CD69 and lacked CD62L (Figure 2e and f), with a fraction of CD69^+^ cells co‐expressing CD103 (Figure 2g and h), a marker associated with tissue residency that is lacking on circulating CD44^+^ T cells.^22^ We observed a significant increase in the number of CD69^−^ and CD69^+^CD103^−/+^ populations within both polyclonal CD8^+^ and SIINFEKL^+^ TIL (Figure 2i and j), suggesting that CD47 × PD‐L1 BisAb treatment promotes a wide‐spread influx of T cells including both tumor‐reactive and polyclonal T cell populations. To next assess the functionality of these cells, CD8^+^ TIL were stimulated with PMA and ionomycin. CD47 × PD‐L1 BisAb treatment significantly enhanced IFNγ production by CD8^+^ T cells compared to isotype treatment, which was significantly greater within the CD69^+^CD103^−/+^ TIL fraction than in the CD69^−^ TIL fraction (Figure 2k and l). Taken together, these results demonstrate that CD47 × PD‐L1 BisAb treatment boosts tumor‐resident CD8^+^ TIL generation and can engender these populations with superior cytokine productivity.

### CD8^+^ T cells play an indispensable role in CD47 × PD‐L1 BisAb‐mediated tumor control

**Figure 3:**
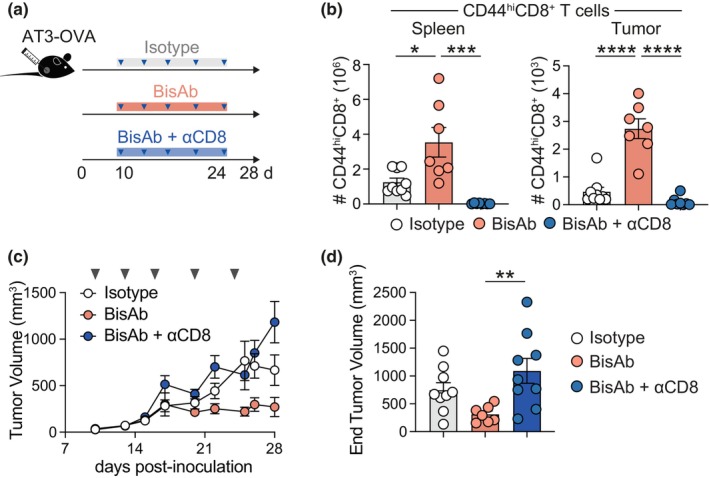
CD47 × PD‐L1 BisAb‐mediated tumor control is dependent on CD8^+^ T cells. Mice were inoculated with AT3‐OVA prior to administration of 10 mg kg^−1^ of anti‐CD8α every 2–3 days from Day 8 post inoculation. On Day 10 post inoculation, mice were treated with 40 mg kg^−1^ of isotype or BisAb i.p and assessed at Day 28. **(a)** Experimental schematic. **(b)** Enumeration of CD44^hi^CD8^+^ T cells in the spleen and tumor. **(c)** Tumor volume over time. **(d)** Tumor volume at experimental endpoint (Day 28). Enumeration of cells in the tumor are expressed per mm^3^. The combined data of two independent experiments with a total of 7 or 8 mice per group is shown. *P* > 0.05, *P ≤ 0.05, **P ≤ 0.01, ***P ≤ 0.001, ****P ≤ 0.0001, one‐way ANOVA with a Kruskal–Wallis post‐test. Bars represent mean ± SEM, symbols represent individual mice.

The observation that CD47 × PD‐L1 BisAb treatment elicited enhanced CD8^+^ TIL formation and function led us to ask whether tumor protection observed upon CD47 × PD‐L1 BisAb treatment was CD8^+^ T cell dependent. To this end, AT3‐OVA inoculated mice were administered anti‐CD8 depleting antibody 2 days prior to the commencement of CD47 × PD‐L1 BisAb or isotype antibody therapy and then treated every 2–3 days thereafter. Anti‐CD8 depleting antibody in conjugation with CD47 × PD‐L1 BisAb treatment resulted in the ablation of CD8^+^ T cells within the spleen and tumor (Figure 3a and b) and led to an outgrowth of AT3‐OVA tumors equivalent to that observed in isotype‐treated mice (Figure 3c and d). We also noted that myeloid populations, including macrophages, monocytes and neutrophils, as well as the CD44^+^ CD4^+^ T cell population, were not altered upon CD47 × PD‐L1 BisAb treatment in the absence of CD8^+^ T cells (Supplementary figure 4). Together, these data suggest a direct role of CD8^+^ T cells in breast cancer tumor regression and that the mechanisms of CD47 × PD‐L1 BisAb‐mediated tumor control are regulated by CD8^+^ T cells.

### CD47 × PD‐L1 BisAb treatment induces superior CD8^+^ T cell responses compared to anti‐PD‐L1 therapy

**Figure 4:**
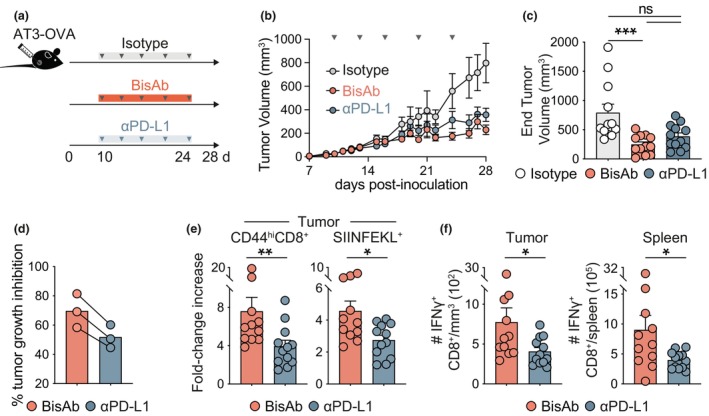
CD47 × PD‐L1 BisAb treatment is superior to anti‐PD‐L1 monotherapy. AT3‐OVA bearing mice were treated with 40 mg kg^−1^ of isotype or BisAb, or 10 mg kg^−1^ of anti‐PD‐L1 i.p every 3–4 days from Day 10 post inoculation before being assessed on Day 28. **(a)** Experimental schematic. **(b)** Tumor volume over time. **(c)** Tumor volume at experimental endpoint (Day 28). **(d)** Tumor growth inhibition expressed as a percentage of endpoint tumor volume measured for the isotype control. Each point represents one independent experiment, where average tumor volume per group was employed to calculate differences between groups. **(e)** Fold‐change increase in enumerated CD44^hi^ and SIINFEKL^+^ CD8^+^ T cells in the tumor from isotype control. **(f)** Enumeration of IFNγ^+^ CD8^+^ T cells in the tumor and spleen upon PMA/Ionomycin stimulation. Enumeration of cells in the tumor are expressed per mm^3^. The combined data of three independent experiments with a total of 10–12 mice per group is shown. ns, *P* > 0.05, *P ≤ 0.05, **P ≤ 0.01, ***P ≤ 0.001, one‐way ANOVA. Bars represent mean ± SEM, symbols represent individual mice.

The results above showed that CD47 × PD‐L1 BisAb treatment promoted both myeloid and T cell infiltration into AT3 tumors, as well as enhanced cytokine production by CD8^+^ TIL. Given our results demonstrating the role of CD8^+^ T cells in CD47 × PD‐L1 BisAb‐mediated tumor control, we assessed the advantage of combined CD47 and PD‐L1 blockade over anti‐PD‐L1 monotherapy. For this, AT3‐OVA‐bearing mice received isotype, CD47 × PD‐L1 BisAb or anti‐PD‐L1 antibody every 3–4 days until the experimental endpoint (Figure 4a). While CD47 × PD‐L1 BisAb and standard anti‐PD‐L1 therapy inhibited tumor growth compared to isotype control (Figure 4b–d), the enhancement of myeloid populations within the spleen was only observed within the CD47 × PD‐L1 BisAb‐treated mice, whereas no changes were observed in the tumor (Supplementary figure 5a and b). Additionally, CD47 × PD‐L1 BisAb treatment was superior in promoting an increase in polyclonal and SIINFEKL^+^ CD8^+^ TIL as compared to anti‐PD‐L1 treatment alone (Figure 4e and Supplementary figure 5c and d). Critically, CD47 × PD‐L1 BisAb treatment resulted in significantly greater IFNγ‐producing CD8^+^ T cells in the tumor and spleen (Figure 4f and Supplementary figure 5e) than anti‐PD‐L1 alone. Altogether, these findings demonstrate that combined targeting of CD47 and PD‐L1 via CD47 × PD‐L1 BisAb is an effective treatment for controlling AT3‐OVA breast cancer and is superior to anti‐PD‐L1 monotherapy for promoting robust CD8^+^ T‐cell responses within the tumor.

## Discussion

Dual targeting of CD47 and PD‐L1 has led to promising preclinical results across tumor models.^16^, ^23^ Engaging both innate and adaptive immune pathways limits resistance and toxicity profiles observed for monotherapies alone or in combination.^24^ Here, we describe the immune landscape generated upon CD47 × PD‐L1 BisAb treatment within the tumor microenvironment and circulation. Our findings revealed a robust enhancement of myeloid cells in the tumor and spleen, along with the development of tumor‐resident CD8^+^ TIL and circulating long‐lived effector T cells that were equipped with heightened pro‐inflammatory cytokine production. In addition to underscoring the pivotal role of CD8^+^ T cells in mediating tumor control, CD47 × PD‐L1 BisAb treatment outperformed anti‐PD‐L1 monotherapy in generating highly functional CD8^+^ TIL, demonstrating the cumulative advantage of reinvigorating adaptive immune responses by disrupting innate inhibitory pathways.

CD47 × PD‐L1 BisAb successfully controlled tumor growth in an orthotopic model of breast cancer and akin to observations in other models, induced the influx of intratumoral macrophages, monocytes and neutrophils.^16^ Nonetheless, tumor growth inhibition by CD47 × PD‐L1 BisAb was primarily mediated by CD8^+^ T cells. Indeed, CD47 × PD‐L1 BisAb greatly augmented antigen‐specific CD8^+^ TIL, which were more capable of secreting IFNγ than their circulating counterparts. This suggests that the clearance of tumor cells and reduced antigen load induced by CD47 × PD‐L1 BisAb may have diverted CD8^+^ TIL differentiation against an exhausted, dysfunctional state. While numerically unchanged upon CD47 × PD‐L1 BisAb treatment, splenic CD8^+^ T cells exhibit higher expression of IFNγ and were skewed towards a long‐lived effector cell phenotype that are shown to be more protective than circulating memory T‐cell phenotypes.^25^, ^26^ It is unclear whether these splenic CD8^+^ T cells are recent emigrants from the tumor or tumor‐draining LN where priming likely occurs,^27^ or whether the pro‐inflammatory conditions generated in the spleen upon CD47 × PD‐L1 BisAb treatment may augment the activation of these cells.^16^ In support of the latter, we observed a skewing of splenic DCs to a cDC1 phenotype, which exhibit heightened transcriptional machinery associated with antigen presentation.^16^

We also noted that tumor‐associated innate populations expressed high levels of CD47 and PD‐L1, suggesting that local CD47 × PD‐L1 BisAb engagement may also regulate CD8^+^ T cell activity. An increase in myeloid populations was not observed following anti‐PD‐L1 monotherapy in the spleen and tumor, supporting the notion that innate and adaptive immune crosstalk may enhance CD8^+^ T cell functionality. Indeed, a combination of increased migrating effector T and myeloid cells as well as modified inflammatory conditions may explain the superior CD8^+^ T‐cell responses mediated by CD47 × PD‐L1 BisAb compared to anti‐PD‐L1 alone. Taken together, these results demonstrate that dual CD47 and PD‐L1 blockade can alter the immune landscape to permit more potent CD8^+^ T cell responses across tissues and inhibit solid tumor growth.

## Methods

### Mice

C57BL/6 mice were bred in the Department of Microbiology and Immunology, The University of Melbourne. Female mice were used at 6–12 weeks of age. All animal experiments were approved by The University of Melbourne Animal Ethics Committee.

### Tumor inoculation

The triple‐negative breast cancer cell line AT3‐OVA was cultured in supplemented Gibco DMEM media (Thermo Fisher Scientific, Waltham, USA) containing 10% FCS (Thermo Fisher Scientific), and once they reached 60–80% confluency, were inoculated into mice as previously described.^19^ Briefly, mice were anaesthetised before injecting 5 × 10^5^ AT3‐OVA cells suspended in 50 μL HBSS into the 4th mammary fat pad. Tumors were measured using a digital calliper, and the volume was estimated using the following formula: volume = [(width^2^ × length)/2]. Tumor growth inhibition was calculated by averaging the end tumor volume per group and comparing the percentage decrease of BisAb‐ or anti‐PD‐L1‐treated mice to isotype‐treated mice within each independent experiment.

### Antibody treatments

Mice used in this study weighed an average of 20 g. Mice were treated with 40 mg kg^−1^ mouse CD47 × PD‐L1 bispecific antibody (Pfizer Inc.^16^); 40 mg kg^−1^ mouse IgG2a isotype control (Leica Biosystems, Wetzlar, Germany) or 10 mg kg^−1^ mouse anti‐PD‐L1 (Clone 10F.6G2, Bio X Cell, Lebanon, USA) via intraperitoneal injection on days 10, 13, 16, 20 and 24 after tumor inoculation. For CD8^+^ cell depletion experiments, 10 mg kg^−1^ of anti‐CD8a (Clone 2.43, Bio X Cell) was administered every 2–3 days to AT3‐OVA bearing mice from Day 8 post‐inoculation.

### Isolation of T cells from mouse tissues and tumors

For flow cytometric analysis, single‐cell suspensions of spleen, mammary fat pad and tumors were prepared. Spleens were isolated by grinding organs through 70‐μm cell strainers and erythrocytes were removed by treatment with red blood cell lysis buffer (Thermo Fisher Scientific, eBioscience). Mammary fat pad and tumors were collected into collagenase III solution (~814 U mL^−1^, Worthington Biochemical, Lakewood, USA) containing DNase I (2.5 μg mL^−1^, Sigma‐Aldrich, St Louis, USA), chopped into fine pieces and incubated for 60 min at 37°C. Digested tissues were passed through a 70‐μm strainer and subjected to red blood cell lysis (Thermo Fisher Scientific, eBioscience).

### Flow cytometry

Single‐cell suspensions were stained with the indicated fluorescently conjugated Ab at 4 °C for 60 min in PBS containing 0.5% FCS and 0.05 m EDTA. Antibodies were validated according to the manufacturer's instructions and are listed in Supplementary table 1. For tetramer staining, cells were incubated with SIINFEKL tetramer (NIH, USA) for at least 45 min on ice prior to surface staining. For cytokine staining, samples were fixed and permeabilised using the BD Cytofix/Cytoperm kit (BD Biosciences, Franklin Lakes, USA) according to the manufacturer's instructions. Cell viability was determined using either fixable Zombie Aqua or Zombie Yellow kits (BioLegend, San Diego, USA). Cells were enumerated by adding SPHERO calibration particles (BD Biosciences) to each sample before acquisition using a Cytek Aurora, and analysis performed using FlowJo (10.6.1; Treestar, Ashland, USA). Representative gating strategies for the populations of interest are shown in Supplementary figure 2, including the following: macrophages (B220^−^NK1.1^−^TCRβ^−^F4‐80^+^), monocytes (F4‐80^−^CD11b^+^Ly6c^hi^), neutrophils (F4‐80^−^CD11b^+^Ly6c^int^), cDC1 (CD11c^+^MHCII^+^ followed by CD8α^+^SIRPα^−^ in spleen, CD103^+^SIRPα^−^ in tumor), cDC2 (CD11c^+^MHCII^+^ followed by CD8α^−^SIRPα^+^ in spleen, CD103^−^SIRPα^+^ in tumor), CD4^+^ T cells (B220^−^NK1.1^−^TCRβ^+^CD44^hi^CD4^+^) and CD8^+^ T cells (B220^−^NK1.1^−^TCRβ^+^CD44^hi^CD8α^+^).

### T cell stimulations

For cytokine profiling, single‐cell suspensions from tissues were cultured in the presence of phorbol myristate acetate (PMA; 50 ng mL^−1^; Sigma‐Aldrich) and ionomycin (1 μg mL^−1^; Sigma‐Aldrich) with Brefeldin A (10 μg mL^−1^; Sigma‐Aldrich) for 4 h before surface staining, fixation and then performing intracellular cytokine staining (BD Cytofix/Cytoperm kit, BD Biosciences).

### Statistical analyses

All experiments were performed at least twice, and data pooled from multiple experiments or display representative flow cytometry plots. Prior to drug and antibody treatments, tumor‐inoculated mice were allocated to groups to ensure equivalent starting mean tumor volumes. Blinding was not required, and appropriate sample sizes were selected to ensure robust statistical testing. All statistical analyses were performed in Prism 8, 9 or 10 (GraphPad, La Jolla, USA), and are indicated in the figure captions. Symbols represent individual mice on the plots, and the mean ± standard error of the mean (SEM) is shown.

## Author contributions

**Susan N Christo** was involved in conceptualisation, design, acquisition, analysis and interpretation of data for the work, intellectual contribution, and editing and writing the manuscript. **Keely M McDonald** was involved in acquisition, analysis and interpretation of data for the work, and editing the manuscript. **Thomas N Burn** was involved in intellectual contribution and editing the manuscript. **Nadia Kurd** was involved in conceptualisation and design of the work, intellectual contribution and editing the manuscript. **Jessica Stanfield** was involved in conceptualisation and design of the work, intellectual contribution and editing the manuscript. **Megan M Kaneda** was involved in editing the manuscript. **Ruth Seelige** and **Christopher P Dillon** were involved in intellectual contribution and editing the manuscript. **Timothy S Fisher** was involved in conceptualisation and design of the work, and editing the manuscript. **Bas Baaten** was involved in conceptualisation and design of the work, intellectual contribution and editing the manuscript. **Laura K Mackay** was involved in conceptualisation, design and interpretation of data for the work, intellectual contribution, and editing and writing the manuscript.

## Conflict of interest

This study was funded by Pfizer Inc. Antibodies presented in this manuscript were developed by Pfizer employees listed as co‐authors.

## Supporting information


Supporting Information

## Data Availability

All data are available from the corresponding author upon reasonable request. Requests for materials should be directed to and will be fulfilled by TSF or BB and the completion of a material transfer agreement with Pfizer.
