# Supplementary material for: Dual CD47 and PD‐L1 blockade elicits anti‐tumor immunity by intratumoral CD8+ T cells
Source: Clin Transl Immunology. 2024 Nov 22;13(11):e70014. doi: 10.1002/cti2.70014 (PMC11583082; doi:10.1002/cti2.70014)
Supplement: Supplementary file 1 — Supporting Information [file CTI2-13-e70014-s001.pdf]

SUPPORTING INFORMATION

Dual CD47 and PD-L1 blockade elicits anti-tumor immunity by intratumoral CD8^{+} T cells

Susan N Christo[1*], Keely M McDonald[1], Thomas N Burn[1], Nadia Kurd[2], Jessica Stanfield[2], Megan M Kaneda, Ruth Seelige[2], Christopher P Dillon[2], Timothy S Fisher[2], Bas Baaten[2] and Laura K Mackay[1,*]

[1]Department of Microbiology and Immunology, The University of Melbourne at the Peter Doherty Institute for Infection and Immunity, Melbourne, VIC, Australia.
[2]Oncology Research Unit, Pfizer Inc., San Diego, CA, USA

*Correspondence should be addressed to S.N.C. (susan.christo@unimelb.edu.au) or L.K.M. (lkmackay@unimelb.edu.au)

Supplementary table 1
Supplementary figures 1-4

Supplementary table 1. List of flow cytometry antibodies used in study.

Reagent	Catalogue Number	Lot Number	Source
Anti-mouse CD103 BV480	566118	1137961	BD Biosciences
Anti-mouse CD103 ef450	48-1031-82	2272751	ThermoFisher
Anti-mouse CD44 BV650	740455	9337711	BD Biosciences
Anti-mouse CD44 BUV395	740215	0237757	BD Biosciences
Anti-mouse CD62L BUV737	612833	0323482	BD Biosciences
Anti-mouse CD69 PECy5	15-0691-82	2265489	Invitrogen
Anti-mouse CD8α BV785	100750	B299807	Biolegend
Anti-mouse CD8α BUV737	612759	0290064	BD Biosciences
Anti-mouse CD8β AF700	126618	B310101	Biolegend
Anti-mouse IFNɣ BV480	566097	0079824	BD Biosciences
Anti-mouse Ly6c BV570	128030	B310124	Biolegend
Anti-mouse TCRβ APC-Cy7	560656	9150630	BD Biosciences
Anti-mouse TCRγδ FITC	553177	8074805	BD Pharmingen
Anti-mouse TNF APC-Cy7	506344	B273382	Biolegend
Anti-mouse TNF BV711	506349	B311216	Biolegend
Anti-mouse B220 BUV661	612972	0170444	BD Biosciences
Anti-mouse NK1.1 BV480	746265	0225254	BD Biosciences
Anti-mouse NK1.1 FITC	553164	8039746	BD Biosciences
Anti-mouse CD4 BUV805	741913	1209823	BD Biosciences
Anti-mouse CD11b BV480	566149	0280243	BD Biosciences
Anti-mouse F4-80 BV421	123137	B323609	Biolegend
Anti-mouse CD11c BV605	117333	B316089	Biolegend
Anti-mouse MHCII AF700	56-5321-82	2210930	Invitrogen
Anti-mouse SIRPα APC	17-1721-82	E14164-104	eBioscience
Anti-mouse SIRPα FITC	560316	2223989	BD Pharmingen
Anti-mouse CD47 PeDazzle 594	127522	B286792	Biolegend
Anti-mouse PD-L1 PE	12-5982-83	EC08317	eBioscience
Anti-mouse CD127 PeDazzle 594	135032	B307880	Biolegend

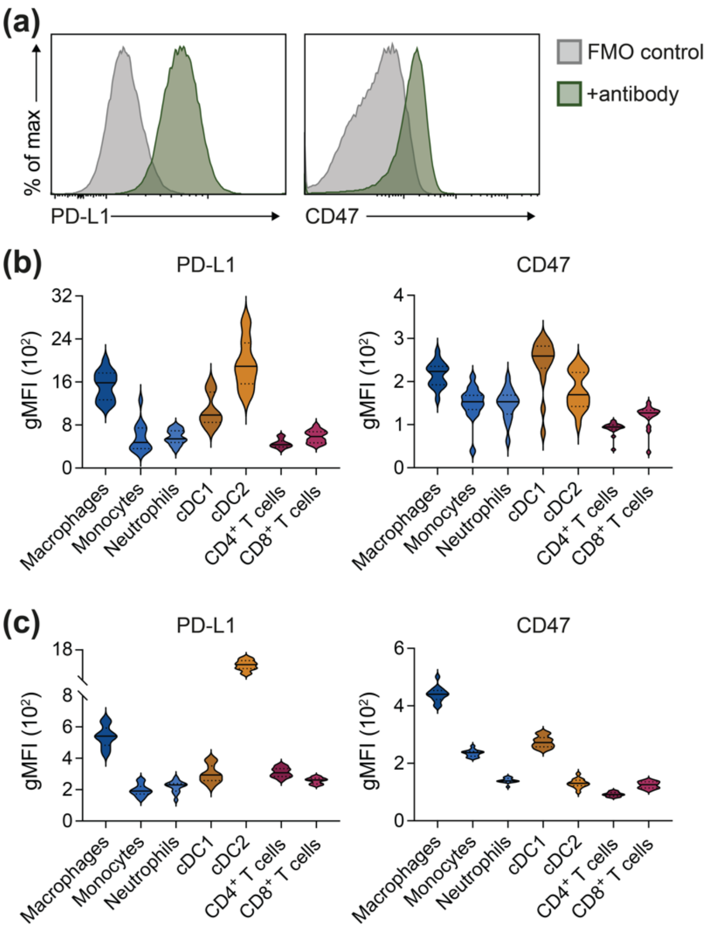

Supplementary figure 1. CD47 and PD-L1 expression by immune populations. (a) Representative contours of CD47 and PD-L1 by AT3-OVA tumor cells *in vitro* compared to fluorescence minus one (FMO) controls (grey). Mice were inoculated with AT3-OVA and 28 days later, the geometric mean fluorescence intensity (gMFI) of CD47 and PD-L1 on indicated cell populations in the tumor **(b)** and spleen **(c)** was assessed. Violin plots represent the distribution of minimum to maximum values. Data represents two independent experiments.

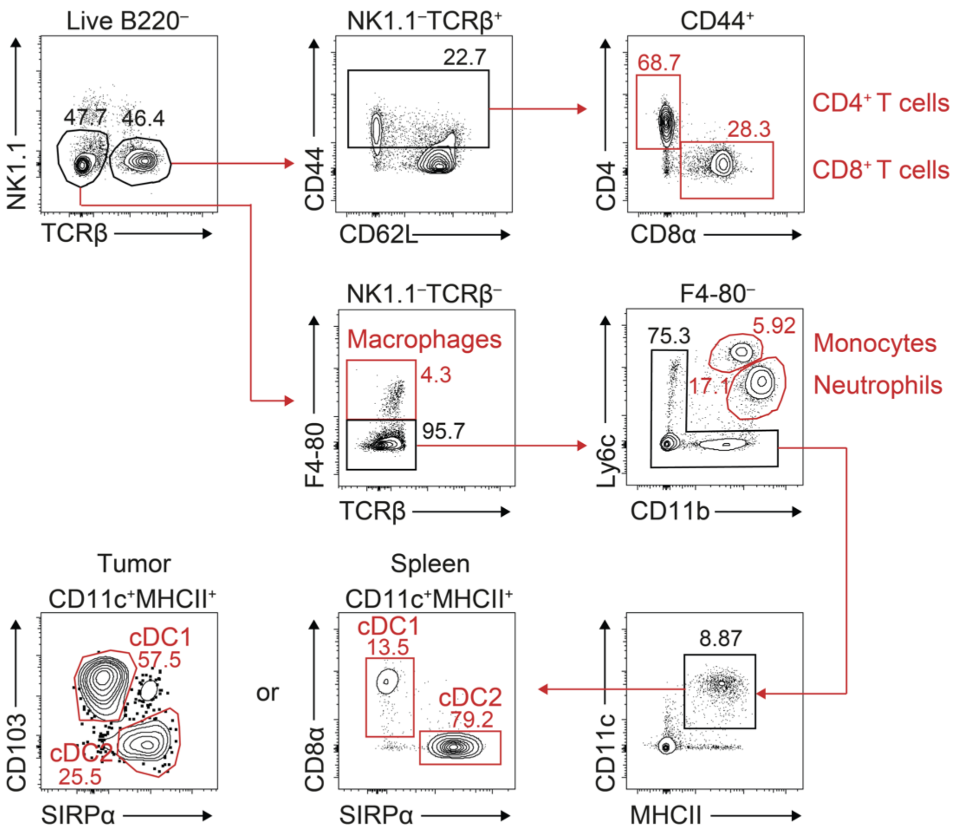

Supplementary figure 2. Gating strategy used to identify immune populations in the spleen and tumor. In spleen and tumor, live cells were excluded of B cells and the resulting B220$^-$ population was then stratified by TCRβ expression that lacked NK1.1 expression (to exclude NK cells and NKT cells). To identify T cells, NK1.1$^-$TCRβ$^+$ cells were gated on CD44^{hi} cells followed by lineage defining markers CD4 and CD8α to identify CD4$^+$ and CD8$^+$ T cells, respectively. Cells that lacked NK1.1 and TCRβ were then gated on F4-80 to identify macrophages, whereas F4-80$^-$ cells expressing CD11b could classify monocytes and neutrophils through Ly6c high and intermediate expression, respectively. The remaining cells (not gated as monocytes or neutrophils) co-expressing CD11c and MHCII were then stratified into cDC1 and cDC2 subsets. In the spleen, cDC1 was defined as CD8α$^+$SIRPα$^-$ and cDC2 was defined as CD8α$^-$SIRPα$^+$. In the tumor, cDC1 was defined as CD103$^+$SIRPα$^-$ and cDC2 was defined as CD103$^-$SIRPα$^+$. The numbers on the plots represent frequencies.

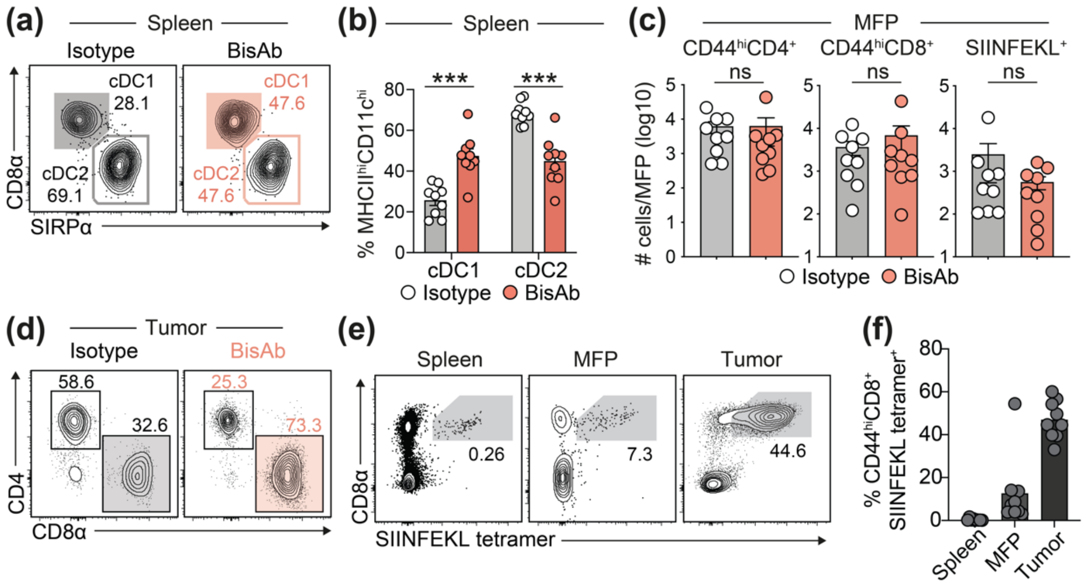

Supplementary figure 3. Effect of CD47×PD-L1 BisAb therapy on immune cells across tissues. (a-d) Mice were orthotopically inoculated with AT3-OVA and treated with 40 mg/kg of isotype or CD47×PD-L1 BisAb i.p from d10 post-inoculation every 3-4 days for a total of five injections before being assessed on d28. **(a)** Representative contour plots and **(b)** frequency of cDC1 ($CD8\alpha^{+}SIRP\alpha^{-}$) and cDC2 ($CD8\alpha^{-}SIRP\alpha^{+}$) in the spleen. **(c)** Enumeration of polyclonal $CD44^{hi}CD4^{+}$ T cells, $CD44^{hi}CD8^{+}$ T cells and $SIINFEKL^{+}$ $CD8^{+}$ T cells in the peritumoral MFP. **(d)** Representative contour plots of CD4 and CD8α expression by $TCR\beta^{+}NK1.1^{-}$ cells in the tumor. **(e)** Representative flow cytometry plots and **(f)** frequency of $SIINFEKL^{+}$ $CD8^{+}$ T cells across tissues. The combined data of two independent experiments with a total of 9 or 10 mice per group is shown. ns, *P* >0.05, **P* ≤0.05, ***P* ≤0.01, ****P* ≤0.001, *****P* ≤0.0001, Mann–Whitney *U*-test. Bars represent mean ± SEM, symbols represent individual mice.

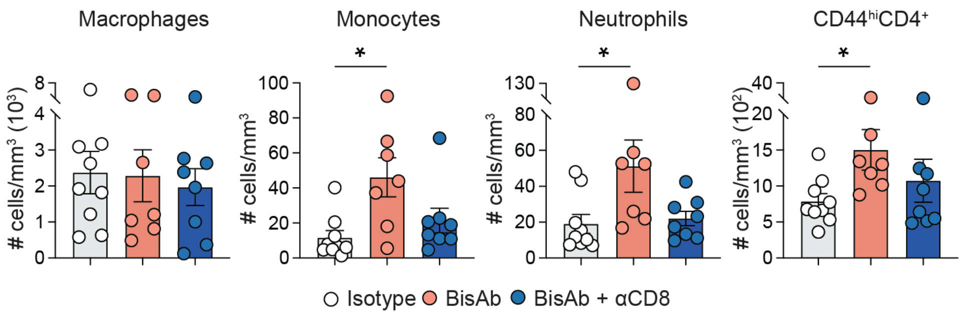

Supplementary figure 4. Effect of CD8^{+} T cell depletion on intratumoral immune populations following CD47×PD-L1 BisAb treatment. Mice were inoculated with AT3-OVA prior to administration of 10 mg/kg of anti-CD8α every 2-3 days from d8 post-inoculation. On d10 post-inoculation, mice were treated with 40 mg/kg of isotype or BisAb i.p and assessed at d28. Enumeration of macrophages, monocytes and neutrophils in the tumor. Enumeration of cells in the tumor are expressed per mm^{3}. The combined data of two independent experiments with a total of 7 or 8 mice per group is shown. ns, *P* >0.05, ***P* ≤0.05, ***P* ≤0.01, ****P* ≤0.001, *****P* ≤0.0001, one-way ANOVA. Bars represent mean ± SEM, symbols represent individual mice.

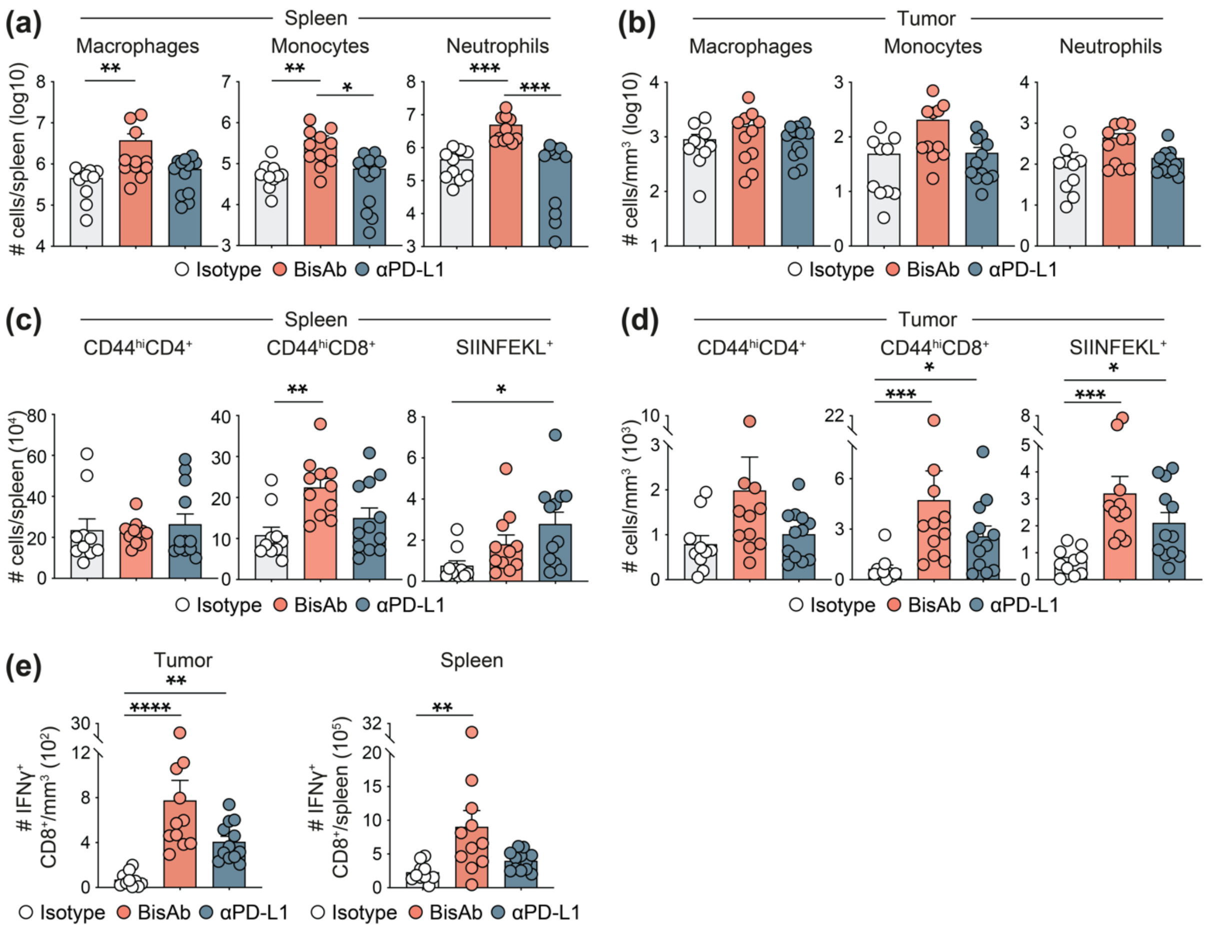

Supplementary figure 5. Dissecting the contribution of CD47 and PD-L1 on immune responses in AT3-OVA tumors. AT3-OVA bearing mice were treated with 40 mg/kg of Isotype or BisAb, or 10 mg/kg of anti-PD-L1 i.p every 3-4 days from d10 post-inoculation before being assessed on d28. Enumeration of macrophages, monocytes and neutrophils in the **(a)** spleen and **(b)** tumor. Enumeration of polyclonal $CD44^{hi}CD4^{+}$ T cells, $CD44^{hi}CD8^{+}$ T cells and $SIINFEKL^{+}$ $CD8^{+}$ T cells in the **(c)** spleen and **(d)** tumor. **(e)** Enumeration of $IFN\gamma^{+}$ $CD8^{+}$ T cells in the tumor and spleen upon PMA/Ionomycin stimulation. Enumeration of cells in the tumor are expressed per mm^3. The combined data of three independent experiments with a total of 10 to 12 mice per group is shown. ns, $P > 0.05$, $*P \leq 0.05$, $**P \leq 0.01$, $***P \leq 0.001$, $****P \leq 0.0001$, one-way ANOVA. Bars represent mean ± SEM, symbols represent individual mice.
